# Removal of Soil Microbes Alters Interspecific Competitiveness of *Epichloë* Endophyte-Infected over Endophyte-Free *Leymus chinensis*

**DOI:** 10.3390/microorganisms8020219

**Published:** 2020-02-06

**Authors:** Hui Liu, Jing Chen, Tianzi Qin, Xinjian Shi, Yubao Gao, Anzhi Ren

**Affiliations:** 1 College of Life Sciences, Nankai University, Tianjin 300071, China; liuhui@mail.nankai.edu.cn (H.L.); chenjing@mail.nankai.edu.cn (J.C.); Tianzi@mail.nankai.edu.cn (T.Q.); xinjian@mail.nankai.edu.cn (X.S.); ybgao@mail.nankai.edu.cn (Y.G.); 2 College of Life Sciences, Dezhou University, Dezhou 253023, China

**Keywords:** *Leymus chinensis*, *Epichloë* endophyte, soil microbe, intraspecific competition, interspecific competition, *Stipa krylovii*

## Abstract

*Epichloë* endophytes may not only affect the growth and resistances of host grasses, but may also affect soil environment including soil microbes. Can *Epichloë* endophyte-mediated modification of soil microbes affect the competitive ability of host grasses? In this study, we tested whether *Epichloë* endophytes and soil microbes alter intraspecific competition between *Epichloë* endophyte-colonized (EI) and endophyte-free (EF) *Leymus chinensis* and interspecific competition between *L. chinensis* and *Stipa krylovii*. The results demonstrated that *Epichloë* endophyte colonization significantly enhanced the intraspecific competitive ability of *L. chinensis* and that this beneficial effect was not affected by soil microbes. Under interspecific competition, however, significant interactions between *Epichloë* endophytes and soil microbes were observed. The effect of *Epichloë* endophytes on interspecific competitiveness of the host changed from positive to neutral with soil microbe removal. Here higher mycorrhizal colonization rates probably contributed to interspecific competitive advantages of EI over EF *L. chinensis*. Our result suggests that *Epichloë* endophytes can influence the competitive ability of the host through plant soil feedbacks from the currently competing plant species.

## 1. Introduction

*Epichloë* endophytes are a group of fungi characterized by their ability to infect the aerial tissues of several cool-season grasses without causing obvious disease [1]. Symbioses between grasses and *Epichloë* endophytes occur in both natural and agricultural grassland communities and are generally considered to be mutualistic [2,3,4]. The host plant provides food, shelter and a mode of reproduction for the fungus, while the fungus can increase host growth, reproduction, and resistance and tolerance to abiotic and biotic stress, such as drought [5,6,7], low nutrients [8,9,10], herbivory [3,11,12] and pathogen attack [13,14].

Given the occurrence of *Epichloë* endophyte-improved plant fitness, *Epichloë* endophytes are assumed to enhance the intra- and interspecific competition ability of host plants [15,16,17,18]. In terms of intraspecific competition, greater shoot and/or root growth in *Festuca arundinacea* [19,20], *Festuca pratensis* [21], and *Bromus benekenii* [22] have been reported in association with the presence of *Epichloë* endophytes. With respect to interspecific competition, the greater competitive ability of plants harbouring *Epichloë* endophytes has been documented in *F. pratensis* [23,24], *Poa alsodes* [25], *Festuca rubra* [16] and *Achnatherum sibiricum* [18]. However, several studies have also reported conflicting results showing neutral to negative effects of endophytes on the competitive ability of the host [26,27,28,29].

*Epichloë* endophytes may not only affect the growth and resistances of host grasses, but may also affect soil microbes, such as nitrogen-fixing bacteria, phosphorus-solubilizing rhizospheric fungi, arbuscular mycorrhizal fungi (AMF) and other microbial communities [30,31,32,33]. Can *Epichloë* endophyte-mediated modification of soil microbes affect the competitive ability of host grasses indirectly? Limited studies focused on AMF. Omacini et al. [15] found that *Epichloë* endophyte colonization reduced AMF colonization of endophyte-infected (EI) *Lolium multiflorum*, while increased AMF colonization of neighbouring endophyte-free (EF) ryegrass. As AMF was neutral to both EI and EF plants, the modified colonization did not change the completive advantage of EI over EF plants. In a recent study, we found that *Epichloë* endophyte colonization significantly inhibited AMF colonization of the host grass *Achnatherum sibiricum*, and the effects of AMF on host competition were variable and depended on the identity of the AMF species [18]. As AMF diversity is high in natural grasslands, and *Epichloë* endophyte colonization can affect AMF as well as other microbes [32,33,34], we hypothesized that *Epichloë* endophyte colonization could not only affect the competitive ability of the host directly by changing its growth and resistance, but also indirectly by changing soil microbes.

*Leymus chinensis* and *Stipa krylovii* are two dominant species of the Inner Mongolia steppe. In the present study, we investigated the effects of *Epichloë* endophytes and soil microbes on the intraspecific competitive interactions between EI and EF *L. chinensis* and interspecific competitive interactions between *L. chinensis* and *S. krylovii*. Specifically, we focused on two primary questions: (1) Does *Epichloë* endophyte colonization affect the growth and intra- and interspecific competitive ability of *L. chinensis*? (2) Is the effect of *Epichloë* endophyte colonization on the growth and competitive ability of *L. chinensis* influenced by soil microbes?

## 2. Materials and Methods

### 2.1. Plant and Fungal Material

**Figure 1:**
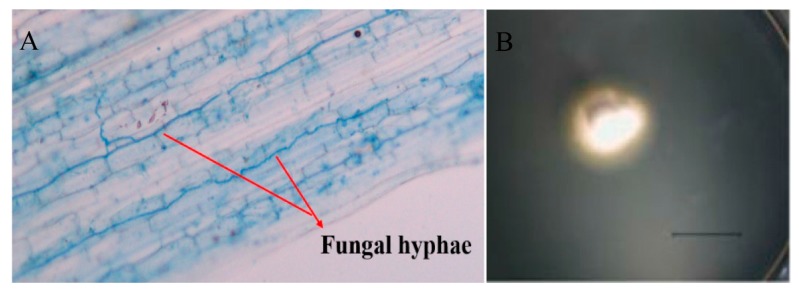
Hyphae in leaf sheath (**A**) (Shot under 4× microscope) and colony (**B**) of *Epichloë bromicola* isolated from *Leymus chinensis*. Picture of colony was taken after growing on PDA for 10 days (scale bars = 10 mm).

*L. chinensis,* a rhizomatous perennial grass, was originally sampled from their natural populations at Abaga Banner in Inner Mongolia (43.90° N, 115.34° E). It can be colonized by the leaf endophyte *Epichloë bromicola*, and the infection rate was about 63% [35]. In this area, *L. chinensis* seldom produces seeds, and stromata have not ever been observed, thus the endophyte is highly likely transmitted via vegetative propagation. EI and EF plants used in this experiment were originally collected from their natural population in summer in 2015, multiplied and selected for uniformity in 2016 and 2017. During this period, we clipped the plants repeatedly and kept them growing vegetatively aiming to avoid possible horizontal transmission of *Epichloë* endophyte, but only vertical transmission between different tillers. The *Epichloë* endophyte status of each *L. chinensis* plant was not only checked microscopically by examining the leaf sheaths for the presence of fungal hyphae after staining with lactophenol aniline blue [36] (Figure 1A) but also isolated (Figure 1B) and identified before conducting experiments to confirm endophyte status.

*S. krylovii*, a perennial bunchgrass, cannot be infected by *Epichloë* endophyte [37]. *S. krylovii* seeds were sampled from their natural populations at Abaga Banner in Inner Mongolia. After nine months of growth in the greenhouse at Nankai University, the plants that were almost the same size as *L. chinensis* were selected.

Soil was collected from the natural grassland hosting the two plant species at Abaga Banner in Inner Mongolia. We sieved the soil samples to remove roots and then pooled them for the experiment. Sterilized soils (which were autoclaved for 90 min at 121 °C) served as a microbe-free (MF) treatment. Non-sterilized soils were used as microbe-inclusive (MI) treatment.

### 2.2. Experimental Design

The experiment had a randomized block design with three factors. One factor, *Epichloë* endophyte colonization status of *L. chinensis*, contained two treatments: EI and EF *L. chinensis*. The second factor, soil microbe status, contained two treatments: MI and MF. These two factors were the same for both intra- and interspecific competition. The difference in the test of the two competition types were the third factor, plant mixture type: monoculture and mixture. The test of intraspecific competition included three groups: monoculture of EI *L. chinensis* (EI), monoculture of EF *L. chinensis* (EF) and mixture of EI and EF *L. chinensis*. In contrast, the test for interspecific competition involved five groups: EI, EF, monoculture of *S. krylovii* (S), mixture of EI *L. chinensis* and *S. krylovii* (EIS) and mixture of EF *L. chinensis* and *S. krylovii* (EFS) (Table 1)

A de Wit-type replacement series [38] with equal plant densities was used to assess the intraspecific competitive interaction between EI and EF *L. chinensis* and the interspecific competitive interaction between *L. chinensis* and *S. krylovii.* Six plant individuals per plant species for the monocultures and three + three individuals of each plant species for the mixtures were transplanted into each plastic pot (22-cm diameter and 17-cm depth) containing 1.5 kg sterilized or non-sterilized soil, resulting in 12 combinations. Each combination was replicated five times, yielding a total of 60 pots. The experiment lasted 4 months, from 12 April 2018 to 12 August 2018, and was conducted in the campus experimental field at Nankai University, Tianjin, China. During the experiment, all pots were watered two or three times a week, and nutrients were supplied by the addition of Hoagland nutrient solution once per week to ensure the normal growth of plants.

### 2.3. Biomass and Relative Yield (RY)

At harvest, the shoots of each species were cut at the soil level, dried at 80 °C for 24 h, and then weighed. Roots were collected by gentle washing, separated according to species and divided into two groups. One group was dried at 80 °C for 24 h and then weighed; the other group was kept at −20 °C for the determination of the AMF colonization rate. Relative yield (RY) was calculated as the ratio of the weight of the species in the mixture to the weight of the species in monoculture.

### 2.4. AMF Colonization Rate

Colonization of the roots by AMF was microscopically assessed. A subsample (ca. 2 g) of roots was cleared with 10% (*w*/*v*) KOH before being stained using 0.05% trypan blue in lactic acid (*v*/*v*) using a modified version of the procedure described in Phillips and Hayman [39]. Mycorrhizal colonization was determined by examining 15–25 1-cm root segments at 200× magnification. The AMF colonization rate was recorded using the cross-hair eyepiece method [40], with a minimum of 200 intersections per replicate.

### 2.5. Competitiveness

The intraspecific competitive ability of EI against EF *L. chinensis* or interspecific competitive ability of *L. chinensis* against *S. krylovii* in the mixture pots was assessed using the aggressivity index (AGR) and the relative interaction intensity index (RII), both of which were calculated using the total biomass of the plant species. The AGR of species x relative to species y was measured according to McGilchrist and Trenbath [41]:

AGR_xy_ = RY_x_ − RY_y_ = (DM_xy_/DM_xx_) (D − M_yx_/DM_yy_) (1)

where RY is the relative yield of species x or y, defined as the dry matter yield of a species grown in mixture (DM_xy_ or DM_yx_) relative to the dry matter in the respective monoculture (DM_xx_ or DM_yy_). If the AGR_xy_ value is zero, then x and y have the same competitive ability. Species x had a higher competitive ability than species y if the AGR_xy_ value was greater than zero. A negative AGR_xy_ value indicated the opposite.

The RII allows for simple comparisons of interaction strength across species and treatments [42]. The RII equation is as follows:

RII = (DM_xy_ − DM_xx_)/(DM_xy_ + DM_xx_) (2)

The RII is a measure of the strength of interactions between species and is centred on zero, with negative interactions (competition) indicated by values between 0 and −1 and positive interactions (facilitation) indicated by values between 0 and +1.

### 2.6. Statistical Analyses

We performed all statistical analyses in the SPSS software (version 20.0, SPSS, Chicago, IL, USA). A two-way ANOVA was conducted to determine the effects of *Epichloë* endophyte colonization (E) and soil microbes (M) on the relative yield, AGR of *L. chinensis* and RII of both *L. chinensis* and *S. krylovii* and to analyse the effects of E and plant mixture type (C) on the mycorrhizal colonization rate of *L. chinensis* and to examine the effects of M and C on the relative yield of *S. krylovii*. We compared the competitiveness of EI relative to EF *L. chinensis* in intraspecific competition and mycorrhizal colonization rate among S, EIS and EFS in interspecific competition, so we only used one-way ANOVA examining the effect of M on the AGR of *L. chinensis* and determining the effect of C on the mycorrhizal colonization rate of *S. krylovii*.

## 3. Results

### 3.1. Plant Growth Performance

**Figure 2:**
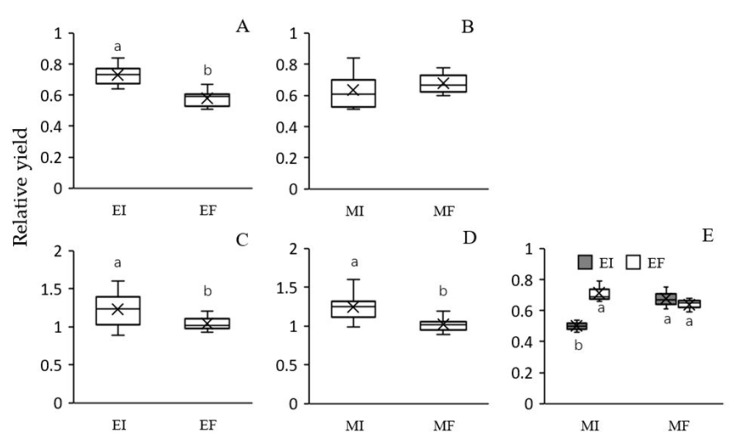
Effects of *Epichloë* endophyte colonization and soil microbes on the relative yield of *Leymus chinensis* both in intraspecific (**A**) and (**B**) and interspecific (**C**) and (**D**) competition and effect of the interaction between soil microbes and plant mixture type on the relative yield of *Stipa krylovii* (**E**). Values are means ± SE (*n* = 5). The different letters above bars denote means that are significantly different among treatments (*p* < 0.05). EI, *Epichloë* endophyte-colonized *L. chinensis*; EF, endophyte-free *L. chinensis*; EIS, *S. krylovii* grown with EI *L. chinensis*; EFS, *S. krylovii* grown with EF *L.chinensis*; MI, soil microbe inclusive; MF, soil microbe free. Symbol ‘×’, horizontal line in the box, top and bottom of box edges and bars indicate the average value, median, 25% and 75% quartiles, and 5% and 95% quantiles, respectively.

Growth of *L. chinensis*, in terms of RY was significantly increased by *Epichloë* endophyte colonization under both intra- and interspecific competition, but the extent of this effect was higher for intraspecific competition (26% versus 18%, respectively) (Table 2, Figure 2A,C). Microbe removal significantly reduced the RY of *L. chinensis* under inter- but not intraspecific competition (Table 2, Figure 2B,D). There was no significant interaction between *Epichloë* endophytes and soil microbes on the RY of *L. chinensis* under either intra- or interspecific competition (Table 2).

The RY of *S. krylovii* was significantly affected by the interaction between soil microbes and plant mixture types (Table 2). In addition, there was no significant effect of *Epichloë* endophytes in *L. chinensis* on the RY of *S. krylovii* in the MF treatment, but *Epichloë* endophyte presence significantly suppressed the RY of *S. krylovii* in the MI treatment (Figure 2E). Thus, *S. krylovii* showed a higher RY when grown with EF than EI *L. chinensis* in the MI treatment (Figure 2E).

### 3.2. AMF Colonization Rate

**Figure 3:**
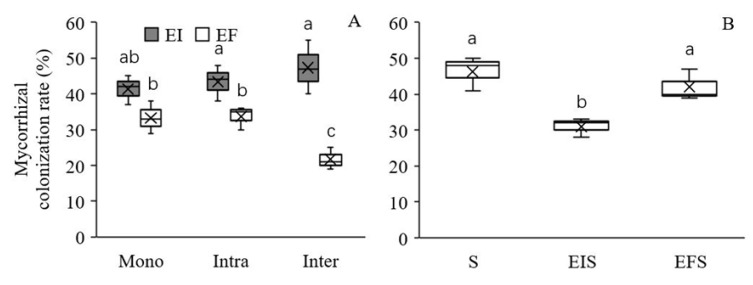
Effect of *Epichloë* endophyte colonization on the mycorrhizal colonization rate of *Leymus chinensis* (**A**) and *Stipa krylovii* (**B**). The different letters above bars denote means that are significantly different among treatments (*p* < 0.05). Mono, monoculture; Intra, intraspecific competition; Inter, interspecific competition; S, the monoculture of *S. krylovii*; EIS, *S. krylovii* grown with EI *L. chinensis*; EFS, *S. krylovii* grown with EF *L. chinensis*. Symbol ‘×’, horizontal line in the box, top and bottom of box edges and bars indicate the average value, median, 25% and 75% quartiles, and 5% and 95% quantiles, respectively.

*Epichloë* endophyte colonization significantly increased the mycorrhizal colonization rate of *L. chinensis* under both intra- and interspecific competition, and this increase was more pronounced under interspecific competition (Table 2, Figure 3A). The mycorrhizal colonization rate of *S. krylovii* was similar in mixtures with EF *L. chinensis* to those in monocultures, which suggests that *L. chinensis* did not affect the mycorrhizal colonization rate of its neighbouring plants. However, the mycorrhizal colonization rate of S. *krylovii* was higher when grown with EF *L. chinensis* plants than when grown with EI plants, which suggests that *Epichloë* endophyte colonization could inhibit the mycorrhizal colonization rate of neighbouring plants (Table 2, Figure 3B).

### 3.3. Competitiveness

**Figure 4:**
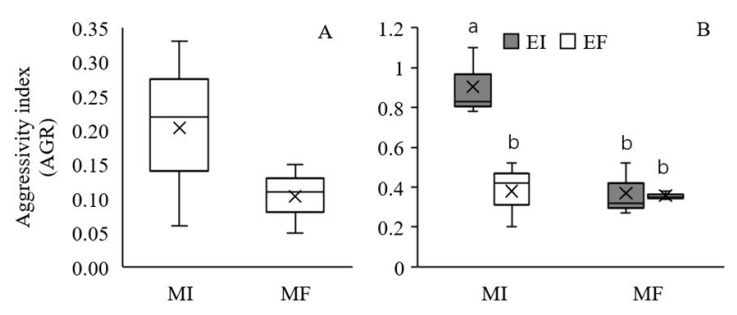
Effect of soil microbes on the aggressivity index (AGR) of *Leymus chinensis* in intraspecific competition (**A**) and effect of the interaction between *Epichloë* endophyte colonization and soil microbes on the AGR of *L. chinensis* in interspecific competition (**B**). The different letters above bars denote means that are significantly different among treatments (*p* < 0.05). EI, *Epichloë* endophyte-colonized *L. chinensis*; EF, endophyte-free *L. chinensis*; MI, soil microbe inclusive; MF, soil microbe free. Symbol ‘×’, horizontal line in the box, top and bottom of box edges and bars indicate the average value, median, 25% and 75% quartiles, and 5% and 95% quantiles, respectively.

The AGR was used to measure the competitive ability of EI *L. chinensis* relative to EF *L. chinensis*. Under intraspecific competition, the AGR of *L. chinensis* was positive in the MI treatment, indicating a greater competitive ability of EI *L. chinensis* relative to that of EF *L. chinensis* (Figure 4A), but no significant main effect of soil microbes was observed (Table 2). Under interspecific competition, the AGR was significantly affected by *Epichloë* endophyte colonization, soil microbes and their interaction (Table 2). *L. chinensis* had a greater competitive ability than *S. krylovii* (AGR > 0). In addition, there was no significant effect of *Epichloë* endophytes on the interspecific competitive ability of *L. chinensis* in the MF treatment, but a higher competitive ability of EI *L. chinensis* compared to EF *L. chinensis* was observed in the MI treatment (Figure 4B).

**Figure 5:**
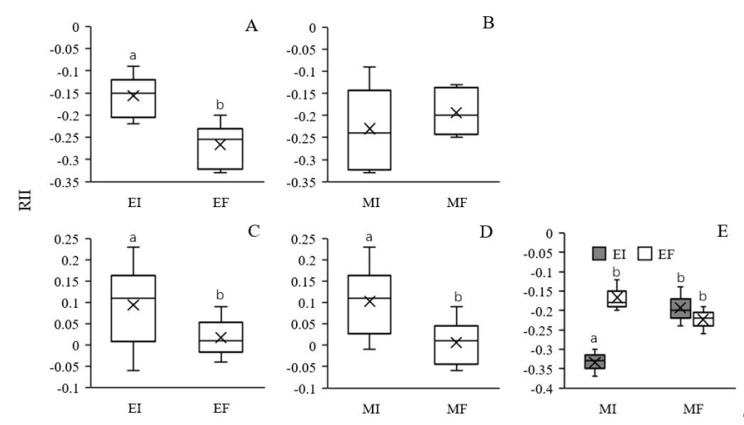
Effects of *Epichloë* endophyte colonization and soil microbes on the relative interaction intensity index (RII) between EI and EF *Leymus chinensis* (**A**) and (**B**) in intraspecific competition and between *L. chinensis* and *Stipa krylovii* for *L. chinensis* in interspecific competition (**C**) and (**D**) and effect of the interaction between *Epichloë* endophyte colonization and soil microbes on the RII between *L. chinensis* and *S. krylovii* for *S. krylovii* in interspecific competition (**E**). The different letters above bars denote means that are significantly different among treatments (*p* < 0.05). EI, *Epichloë* endophyte-colonized *L. chinensis*; EF, endophyte-free *L. chinensis*; MI, soil microbe inclusive; MF, soil microbe free. Symbol ‘×’, horizontal line in the box, top and bottom of box edges and bars indicate the average value, median, 25% and 75% quartiles, and 5% and 95% quantiles, respectively.

The RII provides a simple comparison of interaction strength across species and treatments. Under intraspecific competition, the RII values of both EI and EF *L. chinensis* were negative in both the MI and MF treatments, and only the RII of EI *L. chinensis* was less negative compared to that of EF *L. chinensis* (Table 2, Figure 5A,B). The results indicate that EI and EF *L. chinensis* suppressed each other, but the magnitude of the negative effect of EI on EF *L. chinensis* was larger. The presence of soil microbes did not have a significant effect on the RII of *L. chinensis* (Table 2, Figure 5B). In terms of the response of *L. chinensis* to competition with *S. krylovii*, the presence of *S. krylovii* facilitated the growth of EI *L. chinensis* (RII > 0) but had no influence on EF *L. chinensis*, and a significant main effect of *Epichloë* endophytes was observed (Table 2, Figure 5C). In addition, the presence of soil microbes significantly enhanced the promoting effect of *S. krylovii* on *L. chinensis* under interspecific competition (Table 2, Figure 5D). However, no significant interactive effect between *Epichloë* endophytes and soil microbes was observed (Table 2). The RII of *S. krylovii* was significantly affected by *Epichloë* endophyte colonization, soil microbes and their interaction (Table 2). Furthermore, *L. chinensis* had a negative effect on *S. krylovii* (RII < 0). *Epichloë* endophyte colonization had no significant effect on the RII in the MF treatment, but a stronger negative effect of EI *L. chinensis* on *S. krylovii* was observed in the MI treatment (Figure 5E).

## 4. Discussion

With respect to the effects of *Epichloë* endophyte colonization on plant competitiveness, published studies on the intraspecific or interspecific competitive ability of hosts have focused on different grass-endophyte symbionts. *Epichloë* endophytes can enhance the competitive ability of host plants either by promoting self-growth or by inhibiting the growth of companion plants. For example, Brem and Leuchtman [22] conducted an intraspecific competition experiment and found that *Epichloë* endophytes enhanced the competitive ability of *Br. benekenii* by increasing its aboveground dry matter yield. In relation to the interspecific competition experiment, Craig et al. [25] showed that *Epichloë* endophytes increased the biomass of host *P. alsodes*. Saikkonen et al. [24] also demonstrated that *Epichloë* endophytes conferred a competitive advantage to the host, *F. pratensis*, thereby reducing weed invasions. However, Vázquez-de-Aldana et al. [16] evaluated the effect of *Epichloë* endophyte colonization on the competitive ability of *F. rubra* against five other grassland species in binary mixtures and found that EI plants had better competitive ability than EF plants, as indicated by the lower RY of companion plants when growing in mixture with EI plants. Certainly, neutral [28,29] and negative [26,27] effects of symbiosis when infected plants grow in competition with other species have also been reported. Are the effects of *Epichloë* endophyte colonization on intraspecific and interspecific competition similar for a single grass-endophyte symbiont? The only study that has addressed this question was conducted by Marks et al. [20], who found that *Epichloë* endophyte colonization enhanced both the intra- and interspecific competitive ability of tall fescue but decreased the intra- and interspecific competitive ability of perennial ryegrass. In our study, we found that *Epichloë* endophyte colonization enhanced both the intra- and interspecific competitive ability of *L. chinensis*, only the beneficial effects of *Epichloë* endophyte colonization on the interspecific competitiveness of the host were affected by soil microbes.

In natural systems, plants often experience direct competition with neighbouring plants and simultaneous complex interactions with soil biota [43]. *Epichloë* endophyte colonization can shape communities of soil biota [44,45,46,47,48] and influence subsequent plant growth and survival [49,50,51,52]. Can simultaneous occurrence of foliar *Epichloë* endophytes and soil microbes affect the competitivity of the host grasses? Up to now, only the effects of simultaneous occurrence of foliar *Epichloë* endophytes and AMF were reported and the results were varied. Omacini et al. [15], in binary mixtures of EI and EF *Lolium multiflorum*, found that the competitive ability of plants was increased by the presence of *Epichloë* endophytes, while AMF did not affect host performance in the presence or absence of *Epichloë* endophytes. Vignale et al. [53], in binary mixtures of EI and EF *Bromus auleticus*, showed that neither *Epichloë* endophytes nor AMF affected the intraspecific competitive ability of the plants. In our previor study [18], we found that *Epichloë* endophyte colonization significantly enhanced the interspecific competitive ability of *A. sibiricum*, but there was no interaction between AMF and *Epichloë* endophytes. In the present study, we found that the effect of *Epichloë* endophyte colonization on the intra- and interspecific competitive ability of *L. chinensis* was differentially mediated by soil microbes. Soil microbe presence did not affect the intraspecific competitive ability of EI relative to EF *L. chinensis*. However, with respect to interspecific competition, we did find significant interactions between *Epichloë* endophytes and soil microbes on the competitiveness of *L. chinensis*. The effect of *Epichloë* endophytes on interspecific competitiveness of the host changed from positive to neutral with soil microbe removal.

Why did soil microbes not affect the intraspecific competitive ability but change the interspecific competitive ability of EI over EF *L. chinensis*? In the present study, we investigated AMF colonization rates of *L. chinensis* and *S. krylovii*, and found that AMF colonization rates of EF *L. chinensis* was significantly lower in interspecific competitive studies than those in monoculture and intraspecific competitive studies, while the AMF colonization rates of EI *L. chinensis* did not change significantly. The advantage of EI over EF *L. chinensis* in the mycorrhizal colonization was more evident in interspecific than intraspecific studies. Higher mycorrhizal colonization rates probably contributed to higher interspecific competitive advantages of EI over EF *L. chinensis*.

Plants can shape communities of soil biota and alter soil structure and chemistry in ways that influence subsequent plant growth and survival. The effects of *Epichloë* endophyte colonization on these plant–soil feedbacks (PSFs) have been found both in host grasses as well as in neighbouring plants in the community. Matthew and Clay [50] found that EI *Festuca* had reduced growth in soil that previously supported EI *Festuca*, but was unaffected by soil that once supported EF *Festuca*. Rudgers and Orr [48] reported that soil conditioning by EI tall fescue reduced the biomass of three native tree species via altered soil microbes. In the present study, we found that the effect of *Epichloë* endophytes on interspecific competitiveness changed from positive to neutral with microbe removal. The result suggests that the soil feedback effect of the EI plants can build up quickly during competition. Thus, *Epichloë* endophytes can influence the competitive ability of the host through plant–soil feedbacks from the currently competing plant species.

## Figures and Tables

**Table 1:** The experimental design schematic diagram.

	Intraspecific Competition			Interspecific Competition				
	Monoculture		Mixture	Monoculture			Mixture	
MI	EI	EF	EI and EF	EI	EF	S	EIS	EFS
MF	EI	EF	EI and EF	EI	EF	S	EIS	EFS

EI, *Epichloë* endophyte-colonized *L. chinensis*; EF, endophyte-free *L. chinensis*; EIS, *S. krylovii* grown with EI *L. chinensis*; EFS, *S. krylovii* grown with EF *L. chinensis*; MI, soil microbe inclusive; MF, soil microbe free.

**Table 2:** Analyses of variance (ANOVA) for plant relative yield, mycorrhizal colonization rate, relative interaction intensity index (RII) and aggressivity index (AGR) of *Leymus chinensis* and *Stipa krylovii*.

	Relative Yield				Mycorrhizal Colonization Rate				RII				AGR			
	Intra		Inter		Intra		Inter		Intra		Inter		Intra		Inter	
	*F*	*P*	*F*	*P*	*F*	*P*	*F*	*P*	*F*	*P*	*F*	*P*	*F*	*P*	*F*	*P*
*Leymus chinensis*																
*Epichloë* endophyte (E)	16.573	0.004	6.287	0.037	12.945	0.007	33.228	<0.001	18.441	0.003	5.672	0.044			12.097	0.008
Mycorrhiza (M)	1.362	0.277	9.583	0.015					1.971	0.198	9.557	0.015	1.417	0.300	12.847	0.007
Competition (C)					0.226	0.647	0.941	0.360								
E×M	1.769	0.220	2.915	0.126					2.242	0.173	2.416	0.159			10.713	0.011
E×C					0.115	0.743	9.150	0.016								
M×C																
E×M×C																
*Stipa krylovii*																
*Epichloë* endophyte (E)											7.873	0.023				
Mycorrhiza (M)		2.243	0.173								3.102	0.116				
Competition (C)		6.605	0.033				11.634	0.009								
E×M											16.626	0.004				
M×C		15.096	0.005													

Significant *p*-values (*p* < 0.05). Intra, intraspecific competition; Inter, interspecific competition.
